# 

**Published:** 1907-11-15

**Authors:** 


### ANNOUNCEMENTS.

OHIO STATE BOARD OF DENTAL EXAMINERS.—The regular semi-annual meeting of the Board of Dental Examiners of the State of Ohio, will be held in Columbus, November 26, 27 and 28, 1907.

Only graduates are eligible to examination.

Application, accompanied by fee, $20., should be filed with the Secretary by November 16th. For further information, address, H. C. BROWN, *Secretary*,
185 E. State St., Columbus, Ohio.

THE FORTY-SECOND ANNUAL MEETING OF THE OHIO STATE DENTAL SOCIETY will be held in the assembly rooms of the Great Southern Hotel, Columbus, December 3, 4, and 5, 1907.

An excellent program of papers, clinics and exhibits has been prepared.

The educational features of such a gathering can be appreciated only by those who are in regular attendance at this and other leading societies, and every ethical dentist in the state should come and, if not already a member, should join.

Should you wish to stop at the Great Southern hotel, it would be well to have your rooms reserved in advance, as there are never accommodations for all: however, other first-class hotels are in the immediate vicinity.

Mark the dates off now, and come on the first day, and remain through the entire session.

F. R. CHAPMAN, *Secretary*.

RESOLUTIONS ON THE DEATH OF PROFESSOR MILLER.—

Resolution presented by Holfrat N. S. Jenkins and unanimously adopted by the "W. D. Miller Dental Club," at a special meeting held August 6th.

WHEREAS, Geheim Medicinalrat Professor Dr. Willoughby D. Miller, the great scientist and beloved friend for whom our club was named, has been removed by death; therefore,

RESOLVED: That in common with all the members of our profession, we deeply mourn our irreparable loss.

RESOLVED: That as we were united to him not only by the admiration and respect which his scientific work sp n-taneously evolved, but also by the ties of warm personal friendship, we hereby pledge ourselves to cherish forever the memory of his example, which shall inspire us to higher devotion to our profession, to broader charity, to nobler living and to deeper compassion toward suffering humanity, whom he served so well and so unselfishly.

GEO. O. WEBSTER, *President.*
E. D. BARROWS, *Secretary.*

Berlin, Germany, August 7, 1907.

---

THE INSTITUTE OF DENTAL PEDAGOGIES will hold its next meeting in New Orleans, December 31st, 1907 and January 1st and 2nd, 1908. A good program and a cordial welcome is assured, and all college teachers are urgently requested to attend. The complete announcement and program will be issued as soon as all arrangements are completed.

B. E. LISHER, *Secretary,*
St. Louis, Mo.

---

THE NEW JERSEY STATE BOARD OF REGISTRATION AND EXAMINATION IN DENTISTRY will hold their semi-annual examination beginning Monday, December 9th, and continue through the 10th and 11th. Practical operating and practical prosthetic work will begin 8 a. m., Monday, December 9th. Photograph and preliminary credentials must accompany the application. Meeting held in the State House Trenton, N. J.

For full information address the Secretary.

CHARLES A. MEEKER, D.D.S.,
29 Fulton St., Newark, N. J.

